# The financial burden of out‐of‐pocket healthcare expenses on caregivers of children with atopic dermatitis in the United States

**DOI:** 10.1002/ski2.191

**Published:** 2022-11-20

**Authors:** Raj Chovatiya, Wendy Smith Begolka, Isabelle J. Thibau, Jonathan I. Silverberg

**Affiliations:** ^1^ Department of Dermatology Northwestern University Feinberg School of Medicine Chicago Illinois USA; ^2^ National Eczema Association Novato California USA; ^3^ Department of Dermatology The George Washington University School of Medicine and Health Sciences Washington District of Columbia USA

## Abstract

**Background:**

Atopic dermatitis (AD) is associated with elevated financial costs, including out‐of‐pocket (OOP) expenses. Yet, the full burden of OOP expenses in children with AD is poorly understood.

**Objectives:**

We sought to characterise categories, impact, and associations of caregiver‐reported OOP AD healthcare expenses for US children.

**Methods:**

An online survey was administered to National Eczema Association members (*N* = 113 502). Inclusion criteria (US resident; respondent age ≥18; self or caregiver report of AD diagnosis) was met by 77.3% (1118/1447) of those who completed the questionnaire.

**Results:**

Caregivers of children (<18 years) with AD reported increased healthcare provider (HCP) visits, comorbid food allergy, cutaneous infections, and topical antimicrobial use (*p* < 0.005 for all), and increased OOP expenses for hospitalisation, emergency room visits, emollients, hygiene/bathing products, childcare, and specialised cleaning products, and clothing/bedding (*p* < 0.05 for all) compared to adults with AD. Children with AD had increased median total yearly OOP expenditures ($860 vs. $500, *p* = 0.002) and were more likely to spend ≥$1000 OOP per year (48.9% vs. 40.0%, *p* = 0.03). In children, yearly OOP expenses ≥$1000 were associated with increased AD severity, flares, HCP visits, prescription polypharmacy, and step‐up therapy use (*p* < 0.005 for all) compared with adults. Predictors of harmful financial impact among children included black race (adjusted OR [95% confidence interval]: 3.86 [1.66–8.98] *p* = 0.002) and ≥$1000 annual OOP expenditures (6.98 [3.46–14.08], *p* < 0.0001).

**Conclusion:**

Children with AD have unique and increased OOP expenses that are associated with significant disease burden. Strategies are needed to reduce OOP costs and improve clinical outcomes in children with AD.

## Graphical abstract

Atopic dermatitis (AD) is associated with significant financial costs, including increased out‐of‐pocket (OOP) expenses. OOP healthcare expenses for AD are elevated for caregivers of children and are associated with increased disease activity. Healthcare providers should be mindful of this OOP financial burden strive to maximise clinical outcomes while minimising financial impact.

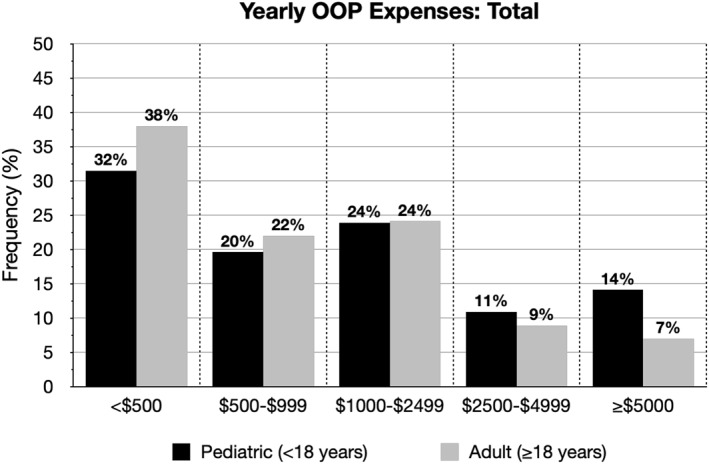

1

**What is already known about this topic?**

- Atopic dermatitis (AD) is associated with significant financial costs, including increased out‐of‐pocket (OOP) expenses.
- The burden of OOP healthcare expenses for management of paediatric AD management are not well understood from the caregiver perspective.

**What does this study add?**

- Caregiver‐reported OOP healthcare expenses for AD in children are elevated versus adults and are associated with increased disease activity.
- Healthcare providers should be mindful of this OOP financial burden and strive to maximise clinical outcomes while minimising financial impact.

## 2 INTRODUCTION

Atopic dermatitis (AD) is a common, chronic inflammatory skin disorder associated with considerable morbidity and quality‐of‐life impairment. AD is especially common in childhood, with current estimates placing the prevalence of AD at 13% among United States (U.S.) children.^1^, ^2^ Heterogeneity of AD, including variable skin manifestations, symptoms, severity, longitudinal course, and comorbidities,^3^ makes AD challenging to treat and burdensome for children, caregivers, and families.^4^, ^5^, ^6^, ^7^

The annual adjusted cost associated with AD management was conservatively estimated to be $5.3 billion USD in 2015. Alongside indirect costs related to time lost from school and work absences, direct costs for AD in children include those associated with increased healthcare resource utilisation, including outpatient healthcare provider (HCP) visits,^8^ inpatient hospitalisations,^9^, ^10^ and urgent care/emergency department visits,^11^ prescription medications,^12^ and variety of other non‐prescription AD‐related healthcare expenditures.^13^, ^14^ Out‐of‐pocket (OOP) costs account for most of these types of expenses and are highly relevant for routine household finances. However, they were difficult to accurately categorise and quantify in previous population and third‐party payer‐based studies given their highly individualised nature.

We recently showed that individuals with AD in the U.S. reported a variety of OOP AD‐related expenditures across outpatient, prescription, non‐prescription therapy, and adjunctive healthcare categories^15^ that were associated with increased AD severity and financial impact.^16^ Little is known about the burden of OOP costs among children with AD. We hypothesised that children with AD had unique increases in various OOP expense categories and overall increased OOP expenses that were associated with increased measures of disease activity and severity. In this study, we determined categories, amount, associations, and impact of OOP expenses for AD in US children.

## 3 METHODS

### 3.1 Study design

Between November and December 2019, the National Eczema Association (NEA; a patient and caregiver advocacy organisation dedicated to improving awareness, clinical care, and research for AD) disseminated a 25‐question online questionnaire to members of NEA, which includes 113 502 unique individuals with AD and non‐affected family members worldwide. Informed consent was obtained electronically prior to questionnaire completion, and those who fully completed were offered the opportunity to enter a randomised drawing for a $50 Amazon gift card. Gift card receipt was not linked in any way to survey responses. Inclusion criteria for the questionnaire were U.S. residency, age ≥18 years, and either personal diagnosis of AD or primary caregiver for a child or adolescent with AD. These inclusion criteria were met by 77.3% (1118/1447) of those who completed the questionnaire.

### 3.2 Questionnaire

AD diagnosis was confirmed by a ‘yes’ answer to ‘Have [you/the person with atopic dermatitis] been diagnosed with atopic dermatitis by a healthcare provider?’ Age, gender, race/ethnicity, insurance coverage, income, and geographic location were among the demographics collected. Medical history included chronic comorbidities. AD‐related questions (answered based on individual report at the time of survey response) included patient‐reported global severity, monthly flare days, perceived disease control, AD‐related HCP visits in the past year, number of AD prescription therapies, and current AD medications. Self‐reported monthly OOP AD expenses for a variety of health‐related categories ($0/$1–50/$51–100/$101–150/$151–200/$201–250/$251–275/$275–300/>$300) were assessed alongside total self‐reported OOP expenses for AD in the past year (free response), OOP expenses in the past month relative to average and overall impact of OOP AD expenses on everyday finances.

### 3.3 Data analysis

Statistical analysis was performed using SAS version 9.4 (SAS Institute). Rao‐Scott chi‐square test was used for categorical variable comparisons. Kruskal‐Wallis test by ranks was used for comparison of median yearly costs. Multivariable logistic regression with backward stepwise selection was used to determine predictors of household financial impact. Post‐hoc correction for multiple dependent tests was performed using the approach of Benjamini‐Hochberg. Corrected *p*‐values ≤0.05 were considered significant.

## 4 RESULTS

### 4.1 Respondent characteristics, disease activity, and treatments

Among the 1118 respondents, 21.4% (*n* = 228) were parents and/or primary caregivers responding on behalf of paediatric AD patients <18 years old. Compared to adults ≥18 years old, paediatric AD patients were more likely to be reported as male sex (40.8% vs. 17.8%), black/African‐American race (14.8% vs. 7.5%), household income ≥$100,000 (38.4% vs. 26.5%), employer‐sponsored (66.5% vs. 55.3%) or Medicaid/state assistance (18.2% vs. 7.5%) insurance, and residence in the east or west south central region (22.6% vs. 13.8%) (*p* < 0.05) (Table S1). Paediatric AD patients had significantly increased caregiver‐reported rates of comorbid food allergy (57.8% vs. 33.6%), frequent/persistent skin infections (26.2% vs. 17.1%) and ≥5 yearly HCP visits for AD (29.7% vs. 20.4%) (*p* < 0.005) with numerically increased rates of severe AD (32.5% vs. 24.9%) (Table S2). In contrast, they also had a higher rate of moderately or very well‐controlled disease (46.5% vs. 33.3%) (*p* < 0.005). Individuals <18 years old were less likely to have caregiver‐reported use of any systemic therapy (34.6% vs. 42.7%) but more likely to have reported use of topical antimicrobials (29.0% vs. 14.9%) or tacrolimus (17.5% vs. 12.0%) (*p* < 0.05) (Table S3).

### 4.2 OOP expenses

#### 4.2.1 Monthly expenses

**FIGURE 1:**
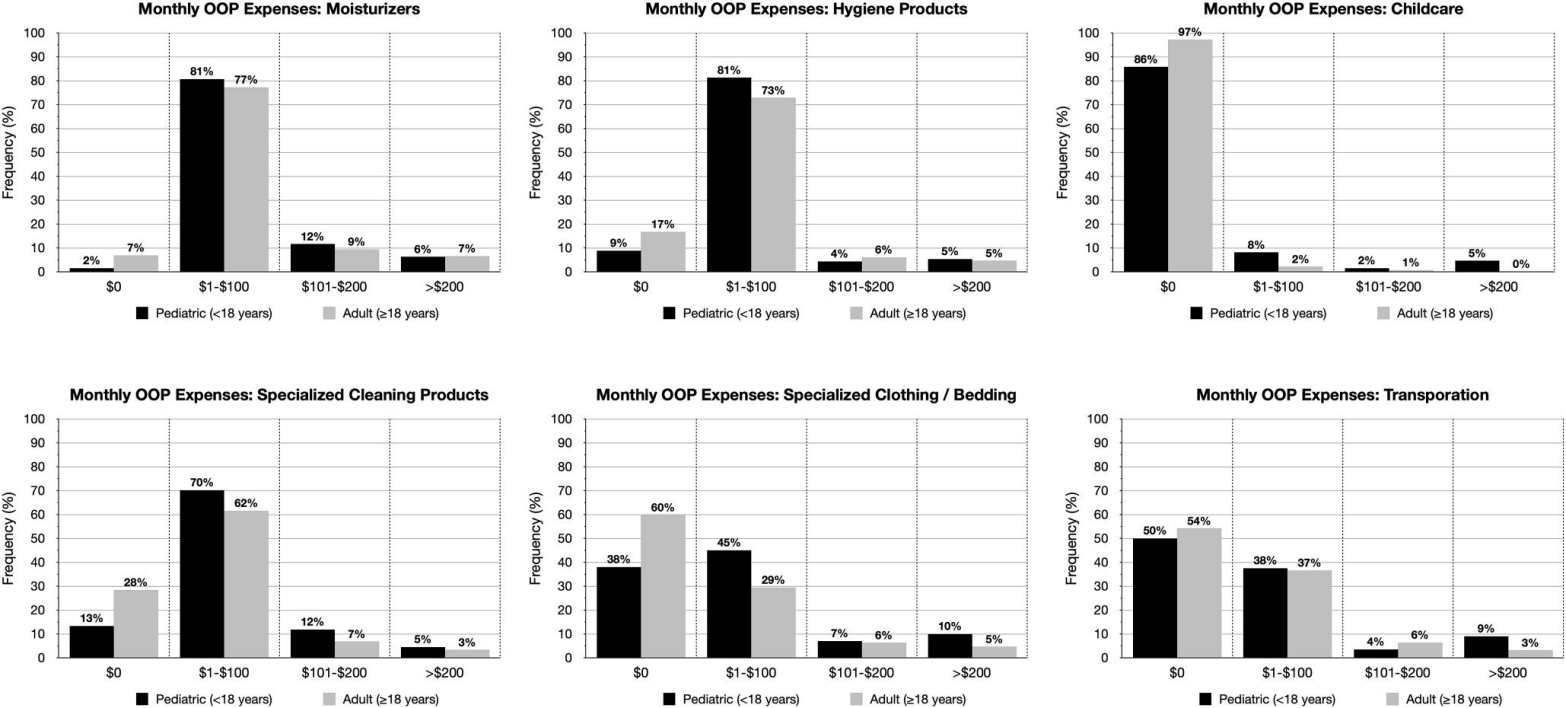
Categories of increased monthly OOP costs in children.

Caregivers of children with AD were more likely to report OOP expenses for several health‐related categories in the past month, including healthcare providers and prescriptions (hospitalisation [<18 vs. ≥18 years old: 4.6% vs. 1.9%], emergency room visits [17.7% vs. 12.1%]), non‐prescription health products (moisturisers [98.6% vs. 93.1%], hygiene products [91.1% vs. 83.3%]), and complementary approaches/care coordination (childcare [14.2% vs. 2.8%], specialised cleaning products [86.6% vs. 71.6%], specialised clothing and bedding [62.0% vs. 40.3%]) (*p* < 0.05) (Figure 1; Table 1). A higher proportion of caregiver respondents reported monthly OOP costs >$100 dollars for moisturisers (17.9% vs. 15.9%), childcare (6.1% vs. 0.7%), specialised cleaning products (16.4% vs. 10.1%), specialised clothing and bedding (17.0% vs. 10.9%), and transportation (12.5% vs. 9.5%). More than half of caregiver respondents described their OOP costs in the past 30 days as significantly or somewhat more (50.4% vs. 38.2%, *p* < 0.05) than their average monthly OOP expenses for AD (Table 2).

**TABLE 1:** Categories of out‐of‐pocket cost by age

	Overall (*n* = 1118)	Age		
Variable—frequency (%)		<18 years (*n* = 228)	≥18 years (*n* = 890)	*p*‐value
Healthcare providers and prescriptions				
Deductible	686 (68.7%)	150 (73.2%)	536 (67.6%)	0.125
Hospitalisation	23 (2.5%)	9 (4.6%)	14 (1.9%)	0.034
Prescriptions covered	635 (64.3%)	133 (65.5%)	502 (64.0%)	0.694
Emergency room visits	123 (13.3%)	35 (17.7%)	88 (12.1%)	0.040
Prescriptions not covered	468 (48.6%)	90 (45.0%)	378 (39.2%)	0.260
Lab testing	216 (23.2%)	49 (25.0%)	167 (22.7%)	0.496
Outpatient phototherapy	42 (4.6%)	4 (2.1%)	38 (5.3%)	0.061
Mental health services	133 (14.4%)	22 (11.2%)	111 (15.3%)	0.144
Non‐prescription health products				
Moisturisers	934 (94.3%)	204 (98.6%)	730 (93.1%)	0.003
Anti‐itch meds	647 (68.3%)	141 (70.5%)	506 (67.7%)	0.441
Allergy meds	715 (75.1%)	151 (75.9%)	564 (74.9%)	0.776
Pain meds	449 (49.3%)	82 (43.2%)	367 (51.0%)	0.055
Sleep meds	336 (37.0%)	50 (25.9%)	286 (40.0%)	0.0003
Bandages	446 (48.4%)	109 (54.5%)	337 (46.7%)	0.052
Hygiene products	824 (85.0%)	185 (91.1%)	639 (83.3%)	0.006
Supplements	491 (52.2%)	94 (47.0%)	397 (53.7%)	0.095
Complementary approaches and care coordination				
Alternative therapy	180 (19.0%)	33 (16.9%)	147 (19.6%)	0.405
Childcare	48 (5.2%)	28 (14.2%)	20 (2.8%)	<0.0001
Adjunctive therapy	150 (15.9%)	11 (5.7%)	139 (18.6%)	<0.0001
Specialised cleaning products	732 (74.7%)	174 (86.6%)	558 (71.6%)	<0.0001
Specialised clothing and bedding	430 (44.8%)	124 (62.0%)	306 (40.3%)	<0.0001
Transportation	444 (46.8%)	100 (50.0%)	344 (45.9%)	0.305

**TABLE 2:** Total out‐of‐pocket (OOP) costs and financial impact by age

	Overall (*n* = 1118)	Age		
Variable		<18 years (*n* = 228)	≥18 years (*n* = 890)	*p*‐value
OOP yearly cost—frequency (%)				
≥$1000	364 (41.9%)	90 (48.9%)	274 (40.0%)	0.030
OOP yearly cost—median (min, max)	600 (0, 200,000)	860 (10, 100 000)	500 (0, 200 000)	0.002
OOP costs in past 30 days reflective of average monthly OOP cost				
Significantly less	51 (5.3%)	11 (5.4%)	40 (5.3%)	0.017
Somewhat less	111 (11.5%)	14 (6.9%)	97 (12.7%)	
Same	410 (42.5%)	76 (37.4%)	334 (43.8%)	
Somewhat more	265 (27.5%)	68 (33.5%)	197 (25.9%)	
Significantly more	128 (13.2%)	34 (16.8%)	94 (12.3%)	
Household financial impact—frequency (%)				
None	61 (6.3%)	8 (3.9%)	53 (7.0%)	0.169
Minimal	281 (29.1%)	54 (26.6%)	227 (29.8%)	
Moderate	387 (40.1%)	85 (41.9%)	302 (39.6%)	
Severe	201 (20.8%)	44 (21.7%)	157 (20.6%)	
Devastating	36 (3.7%)	12 (5.9%)	24 (3.2%)	

#### 4.2.2 Yearly expenses

**FIGURE 2:**
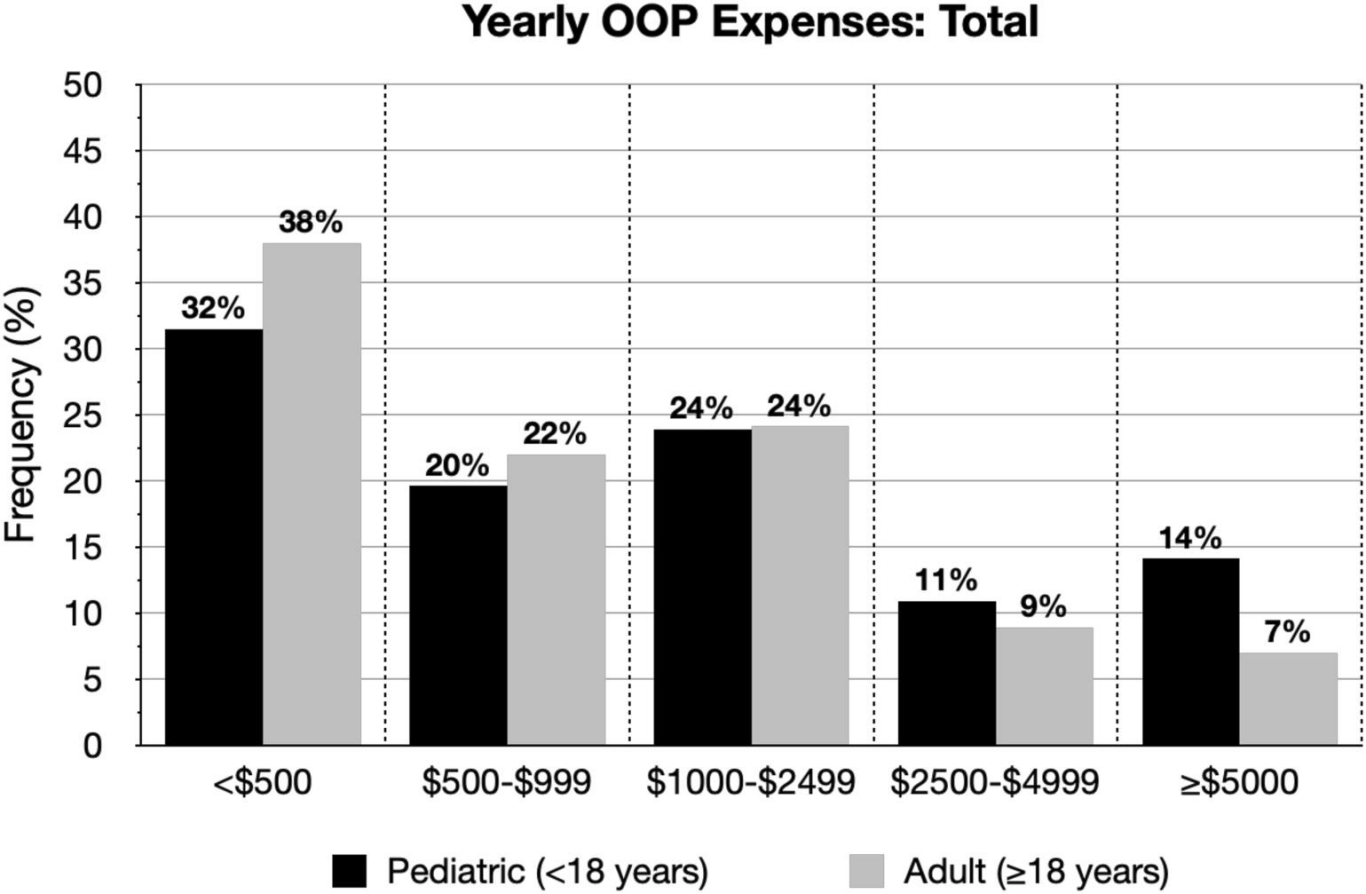
Yearly OOP costs for AD in children and adults.

Caregivers of individuals <18 years of age also experienced higher yearly total OOP costs related to AD care (median [range]: $860 [$10–$100,000] vs. $500 [$0, $200,000], *p* = 0.002), with nearly half reporting OOP costs ≥$1000 (48.9% vs. 40.0%, *p* < 0.05) (Figure 2; Table 2). Those <18 years of age with yearly OOP expenditures of ≥$1000 versus <$1000 were more likely to have moderate and severe disease (45.3% and 69.7%, *p* < 0.0001) (Table 3) They were also more likely to have ≥11 flare days in the past month (67.3%), ≥5 HCP visits for AD in the past year (86.2%), ≥3 prescription treatments (63.4%), and the use of step‐up therapy, that is, systemic therapy including injectable, oral, or phototherapy (69.1%) (*p* < 0.005). While paediatric patients with increased yearly OOP expenses had more caregiver‐reported comorbid allergic rhinitis (60.7%), food allergy (63.8%), and frequent/persistent skin infections (56.4%), they were also numerically more likely to have asthma and anxiety and/or depression.

**TABLE 3:** Associations with total out‐of‐pocket (OOP) costs by age

	Overall (*n* = 1118)	<18 years (*n* = 228)		≥18 years (*n* = 890)	
Variable–frequency (%)	OOP costs ≥$1000/year	OOP costs ≥$1000/year	*p*‐value	OOP costs ≥$1000/year	*p*‐value
Current AD severity					
Clear	6 (28.6%)	2 (40.0%)	<0.0001	4 (25.0%)	<0.0001
Mild	49 (26.5%)	6 (16.7%)		43 (28.9%)	
Moderate	159 (38.5%)	34 (45.3%)		125 (37.0%)	
Severe	144 (62.1%)	46 (69.7%)		98 (59.0%)	
Current AD control					
Minimally controlled	87 (43.5%)	20 (62.5%)	0.284	67 (39.9%)	0.812
Somewhat controlled	155 (43.4%)	35 (48.6%)		120 (42.1%)	
Moderately well controlled	93 (40.8%)	27 (47.4%)		66 (38.6%)	
Very well controlled	28 (36.4%)	8 (36.4%)		20 (36.4%)	
Number of flare days in past 30 days					
0	10 (30.3%)	3 (42.9%)	0.002	7 (26.9%)	0.002
1–3	74 (34.4%)	22 (37.3%)		52 (33.3%)	
4–7	67 (39.9%)	16 (35.5%)		51 (41.5%)	
8–10	44 (36.4%)	14 (70.0%)		30 (29.7%)	
≥11	169 (51.1%)	35 (67.3%)		134 (48.0%)	
Comorbidities					
Asthma	146 (48.8%)	36 (59.0%)	0.054	110 (46.2%)	0.011
Allergic rhinitis	207 (47.5%)	51 (60.7%)	0.003	156 (44.3%)	0.012
Food allergy	185 (55.7%)	67 (63.8%)	<0.0001	118 (52.0%)	<0.0001
Frequent/persistent skin infections	98 (60.1%)	34 (65.4%)	0.005	64 (57.7%)	<0.0001
Anxiety and/or depression	154 (48.4%)	17 (65.4%)	0.070	137 (46.9%)	0.001
HCP visits in past year					
0	12 (15.2%)	1 (7.1%)	<0.0001	11 (16.9%)	<0.0001
1–2	98 (27.5%)	17 (31.5%)		81 (26.8%)	
3–4	116 (50.2%)	22 (37.9%)		94 (54.3%)	
≥5	137 (68.2%)	50 (86.2%)		87 (60.8%)	
Number of treatments					
0	15 (19.2%)	3 (15.8%)	<0.0001	12 (20.3%)	<0.0001
1–2	66 (23.3%)	16 (30.2%)		50 (21.7%)	
≥3	283 (55.7%)	71 (63.4%)		212 (53.5%)	
Step‐up therapy					
No	169 (33.5%)	42 (36.8%)	<0.0001	127 (32.6%)	<0.0001
Yes	190 (53.1%)	47 (69.1%)		143 (49.3%)	

### 4.3 Financial impact

When queried about household financial impacts of OOP AD expenses, most caregivers of paediatric AD patients reported moderate (prevalence [%]: 41.9%), severe (21.7%), or devastating (5.9%) impact (Table 2) A numerically higher proportion in this group also experienced severe or devastating household financial impact versus adults (27.6% vs. 23.8%, respectively). A unique positive predictor of harmful financial impact (i.e., moderate, severe, or devastating) due to OOP AD expense in the paediatric versus adult population was black/African‐American race (adjusted odds ratio [95% confidence interval]: 3.86 [1.66–8.98], *p* = 0.002) (Table 4). Mild disease severity was a unique negative predictor of financial impact (0.11 [0.02–0.81], *p* = 0.03).

**TABLE 4:** Predictors of financial impact

<18 years (*n* = 228)		
Variables	Adjusted odds ratio (95% CI)	*p*‐value
Household income ($)		
≤24,999	1.00 (ref)	–
25,000–99,999	1.49 (0.63–3.52)	0.614
≥100,000	0.50 (0.21–1.20)	0.360
Current AD severity		
Clear	1.00 (ref)	–
Mild	0.11 (0.02–0.81)	0.031
Moderate	0.15 (0.02–1.05)	0.055
Severe	0.34 (0.05–2.37)	0.277
Race		
White	1.00 (ref)	–
American Indian/Alaskan Native	0.09 (0.001–6.33)	0.267
Asian/Native Hawaiian/Pacific Island	2.28 (0.73–7.07)	0.155
Black/African‐American	3.86 (1.66–8.98)	0.002
Multiracial	1.72 (0.64–4.67)	0.285
Other	0.56 (0.06–5.31)	0.614
Annual out‐of‐pocket expenses		
≤$1000	1.00 (ref)	–
>$1000	6.98 (3.46–14.08)	<0.0001

## 5 DISCUSSION

We found that children versus adults had uniquely increased OOP expenses in several different healthcare‐related categories. A greater proportion of children had caregiver‐reported OOP costs for emergency room visits and inpatient hospitalisations. These findings reflect those of U.S. population‐based surveys which showed a high burden of emergency department (ED) encounters and hospitalisations associated with childhood AD.^9^, ^10^, ^11^ Reasons for the high utilisation of inpatient and ED care are multifactorial but likely reflect the overall prevalence of AD in this age group and indicate inadequately controlled disease and limited outpatient access for AD care, especially among those with limited financial resources.^17^ Our data showed poorer disease control and increased allergic and infectious comorbidities among children with AD, despite overall increased yearly HCP visits for AD. These data suggest that additional optimisation of outpatient care is needed in childhood AD.

We found increased OOP expenses for moisturisers, hygiene products, specialised cleaning products, and specialised clothing and bedding. A previous survey of U.S. caregivers revealed an average of $51 spent per month in 2011–2013 on over‐the‐counter (OTC) items for the management of AD, primarily consisting of moisturisers and bath products.^14^ These personal costs were directly associated with emotional impact on children and their families. Another survey of caregivers in the United Kingdom revealed that emollients and bath products were the bulk (>75%) of the £22.03 yearly prescribing cost incurred by the state for AD management in 1995–1996.^18^ Finally, an Australian survey of parents showed that the mean direct cost associated with childhood AD management was tied to disease severity, with major direct costs including dressings, changing of carpets, and new bedding and clothing.^13^ Our data showed that approximately one out of every five caregivers of children with AD spent >$100/month on emollients/moisturisers, specialised cleaning products, and specialised clothing and bedding, while nearly one in 10 spent the same amount on personal hygiene products. These categories of OOP expense are typically not reimbursed by insurance in the US, as they are seen to be ancillary to prescription medical treatment, even though moisturizetion and bathing are foundational, first‐line approaches to AD therapy.^19^ Our data suggest a large financial burden for childhood AD related to many of these OTC products, underscoring the need for HCPs to prioritise flexible, evidence‐based, and fiscally responsible plans when managing non‐medicated approaches. Future efforts to advocate for health plan coverage of basic, non‐prescription therapies for children with AD are critical to reduce the OOP financial burden related to these costs.

Nearly half of caregivers of children with AD experienced OOP costs ≥$1000 per year, and this higher cost was associated with increased severity, flares, HCP visits, prescription therapies, and allergic comorbidities. Similarly, a U.S. population based survey of adults showed that increased OOP costs in AD were associated with poorer overall health and increased HCP office visits.^2^ A French survey of adults with AD showed that OOP expenses are increased among those with more severe AD,^20^ mirroring findings of a larger, multi‐country, European, telephone‐based survey.^21^ Our group recently demonstrated that across all ages in the U.S., increased OOP expenditures were associated with increased overall AD severity, disease flares, poor control, a range of atopic and non‐atopic comorbidities, and prescription medication polypharmacy.^16^ Our findings suggest that caregivers of children with the most active and severe disease shoulder a burden of the OOP costs for AD management. HCPs should pay attention when crafting AD treatment plans for children with highest disease‐burden and aim to appropriately step‐up and step‐down therapy to balance efficacy, safety, and complexity while controlling OOP expenses.

In our study, black race was a unique predictor of harmful household financial impact among children with AD. Previous studies demonstrated that black children have a higher burden of severe AD,^22^ more persistent disease,^23^, ^24^ and increased inpatient^9^ and emergency room^11^ utilisation. We recently showed that black individuals with AD reported more OOP costs for prescription medications, emergency room visits, and experienced higher rates of devasting financial impact related to OOP expenses.^25^ Our findings here further highlight racial disparities in the financial burden of AD. HCPs should be cognisant of this and proactively consider financial impact alongside other elements of the treatment plan.

Strengths of this study include a nationally representative survey of patients with AD and their caregivers. Survey questions addressed multiple patient‐ and caregiver‐centred assessments of disease activity, unique OOP expense categories, and individual financial. The cross‐sectional design of this study is an important limitation as we are unable to analyse longitudinal changes in OOP costs and disease activity. Selection bias is possible given the Internet delivery of the survey tool to NEA members and data being limited to those who responded to the survey and met selection criteria, however, the survey demographics were well represented. Additional studies are needed to confirm these findings and better understand the burden of financial costs related to childhood AD.

## 6 CONCLUSION

In conclusion, caregivers of children with AD experience a distinct financial burden consisting of elevated OOP expenses across a variety of healthcare categories. These OOP costs are associated with significant disease burden. Additional studies are needed to design strategies to reduce OOP costs and improve outcomes in childhood AD.

## CONFLICTS OF INTEREST

Raj Chovatiya has served as an advisory board member, consultant, and/or investigator for AbbVie, Arcutis, Arena, Beiersdorf, Bristol Myers Squibb, Dermavant, Eli Lilly and Company, EPI Health, Incyte, L'Oréal, National Eczema Association, Pfizer Inc., Regeneron, Sanofi, and UCB, and speaker for AbbVie, Dermavant, Eli Lilly and Company, Incyte, Pfizer Inc., Regeneron, Sanofi, and UCB. Wendy Smith Begolka has served as an advisory board member and/or investigator for Incyte and Pfizer. Jonathan Silverberg reports personal fees from Abbvie, Afyx, Arena, Asana, BioMX, Bluefin, Bodewell, Boehringer‐Ingelheim, Celgene, Dermavant, Dermira, Eli Lilly, Galderma, GlaxoSmithKline, Incyte, Kiniksa, Leo, Luna, Menlo, Novartis, Pfizer, RAPT, Regeneron, Sanofi‐Genzyme; institution received grants from Galderma. Isabelle Thibau declares no competing interests.

## AUTHOR CONTRIBUTIONS

**Raj Chovatiya**: Formal analysis (Lead); Validation (Lead); Visualisation (Lead); Writing – original draft (Lead). **Wendy Smith Begolka**: Conceptualisation (Lead); Investigation (Lead); Methodology (Equal); Project administration (Lead); Supervision (Lead); Writing – review & editing (Equal). **Isabelle J. Thibau**: Conceptualisation (Supporting); Data curation (Lead); Investigation (Supporting); Methodology (Supporting); Writing – review & editing (Equal). **Jonathan I. Silverberg**: Conceptualisation (Equal); Formal analysis (Equal); Methodology (Equal); Visualisation (Equal); Writing – review & editing (Equal).

## ETHICAL STATEMENT

Not applicable.

## Supporting information

Supplementary Information S1

## Data Availability

Research data are not shared.
