# Supplementary material for: The financial burden of out‐of‐pocket healthcare expenses on caregivers of children with atopic dermatitis in the United States
Source: Skin Health Dis. 2022 Nov 20;3(1):e191. doi: 10.1002/ski2.191 (PMC9892453; doi:10.1002/ski2.191)
Supplement: Supplementary file 1 — Supplementary Information S1 [file SKI2-3-e191-s001.docx]

| **Supplementary Table 1: Respondent characteristics by age** | | | | |
| --- | --- | --- | --- | --- |
|  | **Overall (n=1,118)** | **Age** | | |
| **Variable – frequency (%)** |  | **<18 years**  **(n=228)** | **≥18 years**  **(n=890)** | **P-value** |
| Gender |  |  |  |  |
| Female | 855 (76.5%) | 133 (58.3%) | 722 (81.1%) | <0.0001 |
| Male | 251 (22.5%) | 93 (40.8%) | 158 (17.8%) |  |
| Non-binary/other | 4 (0.4%) | 0 (0.0%) | 4 (0.5%) |  |
| Prefer not to answer | 8 (0.7%) | 2 (0.9%) | 6 (0.7%) |  |
| Race |  |  |  |  |
| American Indian / Alaskan Native | 8 (0.8%) | 2 (1.0%) | 6 (0.8%) | 0.016 |
| Asian | 58 (6.0%) | 14 (6.9%) | 44 (5.8) |  |
| Black / African-American | 102 (10.6%) | 30 (14.8%) | 72 (7.5%) |  |
| Multiracial | 63 (6.5%) | 21 (10.3%) | 42 (5.5%) |  |
| Native Hawaiian / Pacific Islander | 7 (0.7%) | 0 (0.0%) | 7 (0.9%) |  |
| Other | 28 (2.9%) | 3 (1.5%) | 25 (3.3%) |  |
| White | 697 (72.4%) | 133 (65.5%) | 564 (74.2%) |  |
| Household income ($) |  |  |  |  |
| ≤24,999 | 175 (18.3%) | 36 (17.7%) | 139 (18.5%) | 0.001 |
| 25,000-49,999 | 190 (19.9%) | 22 (10.8%) | 168 (22.4%) |  |
| 50,000-74,999 | 192 (20.1%) | 47 (23.2%) | 145 (19.3%) |  |
| 75,000-99,999 | 122 (12.8%) | 20 (9.9%) | 102 (13.6%) |  |
| 100,000-124,999 | 103 (10.8%) | 25 (12.3%) | 78 (10.4%) |  |
| 125,000-149,999 | 61 (6.4%) | 17 (8.4%) | 44 (5.9%) |  |
| ≥150,000 | 111 (11.6%) | 36 (17.7%) | 75 (10.0%) |  |
| Insurance |  |  |  |  |
| None | 41 (4.3%) | 3 (1.5%) | 38 (5.1%) | <0.0001 |
| Employer-sponsored coverage | 550 (57.7%) | 135 (66.5%) | 415 (55.3%) |  |
| Medicaid or state assistance | 93 (9.8%) | 37 (18.2%) | 56 (7.5%) |  |
| Medicare | 160 (16.8%) | 6 (3.0%) | 154 (20.5%) |  |
| Policy purchased on state/federal health exchange | 37 (3.9%) | 6 (3.0%) | 31 (4.1%) |  |
| Policy purchased on the commercial market | 29 (3.0%) | 6 (3.0%) | 23 (3.1%) |  |
| Tricare or VA benefit | 22 (2.3%) | 7 (3.5%) | 15 (2.0%) |  |
| Unsure | 22 (2.3%) | 3 (1.5%) | 19 (2.5%) |  |
| Geographical setting |  |  |  |  |
| Urban | 229 (23.8%) | 41 (20.3%) | 147 (19.4%) | 0.907 |
| Suburban | 544 (56.6%) | 115 (56.9%) | 429 (56.2%) |  |
| Rural | 188 (19.6%) | 46 (22.8%) | 183 (24.1%) |  |
| Region |  |  |  |  |
| New England | 65 (6.8%) | 17 (8.4%) | 48 (6.3%) | 0.029 |
| Mid-Atlantic | 128 (13.3%) | 18 (8.9%) | 110 (14.5%) |  |
| East North Central | 145 (15.1%) | 28 (13.8%) | 117 (15.4%) |  |
| West North Central | 53 (5.5%) | 10 (4.9%) | 43 (5.7%) |  |
| South Atlantic | 185 (19.2%) | 39 (19.2%) | 146 (19.2%) |  |
| East South Central | 61 (6.3%) | 23 (11.3%) | 38 (5.0%) |  |
| West South Central | 90 (9.4%) | 23 (11.3%) | 67 (8.8%) |  |
| Mountain | 74 (7.7%) | 13 (6.4%) | 61 (8.0%) |  |
| Pacific | 162 (16.8%) | 32 (15.8%) | 130 (17.1%) |  |

| **Supplementary Table 2: Respondent disease burden by age** | | | | |
| --- | --- | --- | --- | --- |
|  | **Overall (n=1,118)** | **Age** | | |
| **Variable – freq (%)** |  | **<18 years**  **(n=228)** | **≥18 years**  **(n=890)** | **P-value** |
| Current AD severity |  |  |  |  |
| Clear | 29 (2.6%) | 5 (2.2%) | 24 (2.7%) | 0.088 |
| Mild | 238 (21.3%) | 51 (22.4%) | 187 (21.0%) |  |
| Moderate | 531 (47.5%) | 96 (42.1%) | 435 (48.9%) |  |
| Severe | 296 (26.5%) | 74 (32.5%) | 222 (24.9%) |  |
| Current AD control |  |  |  |  |
| Minimally controlled | 259 (23.2%) | 38 (16.7%) | 221 (24.8%) | 0.003 |
| Somewhat controlled | 448 (40.1%) | 83 (36.4%) | 365 (41.0%) |  |
| Moderately well controlled | 300 (26.8%) | 77 (33.8%) | 223 (25.1%) |  |
| Very well controlled | 102 (9.1%) | 29 (12.7%) | 73 (8.2%) |  |
| Number of flare days in past 30 days |  |  |  |  |
| 0 | 44 (4.0%) | 10 (4.5%) | 34 (3.8%) | <0.0001 |
| 1-3 | 271 (24.4%) | 78 (34.8%) | 193 (21.8%) |  |
| 4-7 | 206 (18.6%) | 49 (21.9%) | 157 (17.7%) |  |
| 8-10 | 151 (13.6%) | 26 (11.6%) | 125 (14.1%) |  |
| ≥11 | 437 (39.4%) | 61 (27.2%) | 376 (42.5%) |  |
| Comorbidities |  |  |  |  |
| Asthma | 382 (34.5%) | 76 (33.8%) | 306 (34.7%) | 0.788 |
| Allergic rhinitis | 557 (50.4%) | 99 (44.0%) | 458 (52.0%) | 0.033 |
| Food allergy | 426 (38.5%) | 130 (57.8%) | 296 (33.6%) | <0.0001 |
| Frequent/persistent skin infections | 210 (19.0%) | 59 (26.2%) | 151 (17.1%) | 0.002 |
| Anxiety and/or depression | 404 (36.5%) | 32 (14.2%) | 372 (42.2%) | <0.0001 |
| Healthcare provider visits in past year |  |  |  |  |
| 0 | 113 (10.6%) | 16 (7.3%) | 97 (11.4%) | 0.004 |
| 1-2 | 435 (40.6%) | 74 (33.8%) | 361 (42.4%) |  |
| 3-4 | 284 (26.5%) | 64 (29.2%) | 220 (25.8%) |  |
| ≥5 | 239 (22.4%) | 65 (29.7%) | 174 (20.4%) |  |
| Total number of prescription treatments |  |  |  |  |
| 0 | 112 (10.4%) | 23 (10.5%) | 89 (10.4%) | 0.409 |
| 1-2 | 344 (32.1%) | 70 (32.0%) | 274 (32.1%) |  |
| 3-4 | 311 (29.0%) | 55 (25.1%) | 256 (30.0%) |  |
| ≥5 | 306 (28.5%) | 71 (32.4%) | 235 (27.5%) |  |

| **Supplementary Table 3: Respondent treatments by age** | | | | |
| --- | --- | --- | --- | --- |
|  | **Overall (n=1,118)** | **Age** | | |
| **Variable – frequency (%)** |  | **<18 years**  **(n=228)** | **≥18 years**  **(n=890)** | **P-value** |
| Systemic Therapy |  |  |  |  |
| Any | 442 (41.0%) | 75 (34.6%) | 367 (42.7%) | 0.030 |
| Antimicrobials, oral | 80 (7.5%) | 18 (8.3%) | 62 (7.2%) | 0.586 |
| Azathioprine | 12 (1.1%) | 1 (0.5%) | 11 (1.3%) | 0.305 |
| Corticosteroids, injectable | 53 (5.0%) | 11 (5.1%) | 43 (5.0%) | 0.967 |
| Corticosteroids, oral | 110 (10.3%) | 20 (9.2%) | 92 (10.7%) | 0.523 |
| Cyclosporine | 31 (2.9%) | 7 (3.2%) | 24 (2.8%) | 0.732 |
| Dupilumab | 148 (13.9%) | 22 (10.1%) | 130 (15.1%) | 0.061 |
| Methotrexate | 20 (1.9%) | 2 (0.9%) | 18 (2.1%) | 0.253 |
| Mycophenolate Mofetil | 4 (0.4%) | 0 (0.0%) | 4 (0.5%) | 0.314 |
| Phototherapy | 56 (5.3%) | 5 (2.3%) | 53 (6.2%) | 0.024 |
| Tacrolimus | 35 (3.3%) | 7 (3.2%) | 29 (3.4%) | 0.915 |
| Topical Therapy |  |  |  |  |
| Any | 1061 (99.7%) | 217 (100.0%) | 856 (99.5%) | 0.314 |
| Antimicrobials | 190 (17.9%) | 63 (29.0%) | 128 (14.9%) | <0.0001 |
| Corticosteroids | 803 (75.3%) | 168 (77.4%) | 643 (74.5%) | 0.375 |
| Crisaborole | 130 (12.2%) | 30 (13.8%) | 102 (11.9%) | 0.430 |
| Pimecrolimus | 88 (8.3%) | 19 (8.8%) | 71 (8.3%) | 0.812 |
| Tacrolimus | 138 (13.0%) | 38 (17.5%) | 103 (12.0%) | 0.030 |
